# Ablation of STAT3 in the B Cell Compartment Restricts Gammaherpesvirus Latency *In Vivo*

**DOI:** 10.1128/mBio.00723-16

**Published:** 2016-08-02

**Authors:** Sandeep Steven Reddy, Hui-Chen Chang Foreman, Thubten Ozula Sioux, Gee Ho Park, Valeria Poli, Nancy C. Reich, Laurie T. Krug

**Affiliations:** a Department of Molecular Genetics and Microbiology, Stony Brook University, Stony Brook, New York, USA; b Department of Molecular Biotechnology and Health Sciences, University of Torino, Turin, Italy

## Abstract

A challenging property of gammaherpesviruses is their ability to establish lifelong persistence. The establishment of latency in B cells is thought to involve active virus engagement of host signaling pathways. Pathogenic effects of these viruses during latency or following reactivation can be devastating to the host. Many cancers, including those associated with members of the gammaherpesvirus family, Kaposi’s sarcoma-associated herpesvirus and Epstein-Barr virus, express elevated levels of active host signal transducer and activator of transcription-3 (STAT3). STAT3 is activated by tyrosine phosphorylation in response to many cytokines and can orchestrate effector responses that include proliferation, inflammation, metastasis, and developmental programming. However, the contribution of STAT3 to gammaherpesvirus pathogenesis remains to be completely understood. This is the first study to have identified STAT3 as a critical host determinant of the ability of gammaherpesvirus to establish long-term latency in an animal model of disease. Following an acute infection, murine gammaherpesvirus 68 (MHV68) established latency in resident B cells, but establishment of latency was dramatically reduced in animals with a B cell-specific STAT3 deletion. The lack of STAT3 in B cells did not impair germinal center responses for immunoglobulin (Ig) class switching in the spleen and did not reduce either total or virus-specific IgG titers. Although ablation of STAT3 in B cells did not have a global effect on these assays of B cell function, it had long-term consequences for the viral load of the host, since virus latency was reduced at 6 to 8 weeks postinfection. Our findings establish host STAT3 as a mediator of gammaherpesvirus persistence.

## IMPORTANCE

The insidious ability of gammaherpesviruses to establish latent infections can have detrimental consequences for the host. Identification of host factors that promote viral latency is essential for understanding latency mechanisms and for therapeutic interventions. We provide the first evidence that STAT3 expression is needed for murine gammaherpesvirus 68 to establish latency in primary B cells during an active immune response to infection. STAT3 deletion in B cells does not impair adaptive immune control of the virus, but loss of STAT3 in B cells has a long-lasting impact on viral persistence. These results indicate a potential therapeutic benefit of STAT3 inhibitors for combating gammaherpesvirus latency and, thereby, associated pathologies.

## INTRODUCTION

Pathogens that cause chronic disease such as herpesviruses are a challenge to treat and eradicate because they use latency as a strategy of persistence in the host. Most gammaherpesviruses target B lymphocytes as a latency reservoir, ultimately establishing an immunologically silent form of persistence with minimal viral gene expression (1, 2). Viral gene expression during latency can promote lymphoproliferative disease, and lytic reactivation from latent reservoirs can also lead to severe pathologies. It is imperative to identify not only viral determinants but also host determinants that support gammaherpesvirus latency in order to develop novel interventions. Infections by the murine gammaherpesvirus 68 (MHV68) pathogen recapitulate many aspects of human gammaherpesvirus infection, including B cell tropism, long-term establishment of latency in class-switched B cells of the host, and a propensity for lymphomagenesis following impairment of adaptive immune control (2, 3). This model pathogen system affords an analysis of the molecular determinants of latency during the course of a natural host infection.

Signal transducer and activator of transcription 3 (STAT3) is classically activated by tyrosine phosphorylation in response to Janus kinases associated with cytokine receptors (4–6). It is a major downstream target of the interleukin-6 (IL-6) and IL-10 families of cytokines, interferons, growth factors, and oncogenic tyrosine kinases, and it functions as a transcription factor that binds consensus sequences in the regulatory regions of nuclear genes. Constitutive STAT3 activation is associated with oncogenesis (7–10). STAT3 signaling is also stimulated by human gammaherpesvirus gene products such as Kaposi’s sarcoma-associated herpesvirus (KSHV) viral IL-6 (vIL-6) (11–14), kaposin B (15), and viral-G-protein-coupled receptor (v-GPCR) (16, 17) and Epstein-Barr virus (EBV) LMP-1 (18, 19) and EBNA2 (20); and STAT3 levels influence lytic activation of these viruses in cell culture (21–23). Characterized effector responses of STAT3 include survival and proliferation via upregulation of *bcl-2* and c*-myc*, respectively, and promotion of epithelial-mesenchymal transition via Twist (7, 24, 25).

The effect of STAT3 is difficult to predict solely on the basis of putative DNA binding sites. STAT3 binds nonconsensus DNA elements and complexes with transcription factors, including other STATs and NF-κB subunits, and STAT3 functions in phosphorylated and unphosphorylated forms to direct changes in gene expression and chromatin remodeling in a cell-specific manner (4, 26). Nuclear-cytoplasmic shuttling of STAT3 is independent of its phosphorylation status (27); however, STAT3-protein interactions and gene regulation are influenced by posttranslational modifications, in particular, tyrosine and serine phosphorylation (4, 28, 29). STAT3 has also been shown to have nongenomic effects (30, 31).

In the natural course of infection, primary B cells that are infected with a gammaherpesvirus are influenced by the cytokine and cellular interactions in the microenvironment of lymphoid tissues (32–34). Thus, to understand the biological role of STAT3 in shaping gammaherpesvirus latency, an examination of pathogenesis in the whole animal is required. We used MHV68 to examine the impact of STAT3 loss on chronic gammaherpesvirus infection in a natural rodent host. Since the STAT3 murine knockout is embryonically lethal, we used tissue-specific deletion of STAT3 with the Cre-lox recombination system (35). Mice with a B cell-specific deletion of STAT3 (CD19-Cre) were infected with MHV68. We found that MHV68 requires STAT3 to establish latency irrespective of the route of infection and that this intrinsic requirement for STAT3 is not linked to dysfunction in T cell-dependent B cell processes. The latency defect in STAT3-null B cells was sustained for up to 8 weeks after infection. Thus, we conclude that STAT3 is a critical host determinant at both early and late stages of a chronic gammaherpesvirus infection.

## RESULTS

### Deletion of *stat3* from B cells impairs establishment of gammaherpesvirus latency.

**FIG 1:**
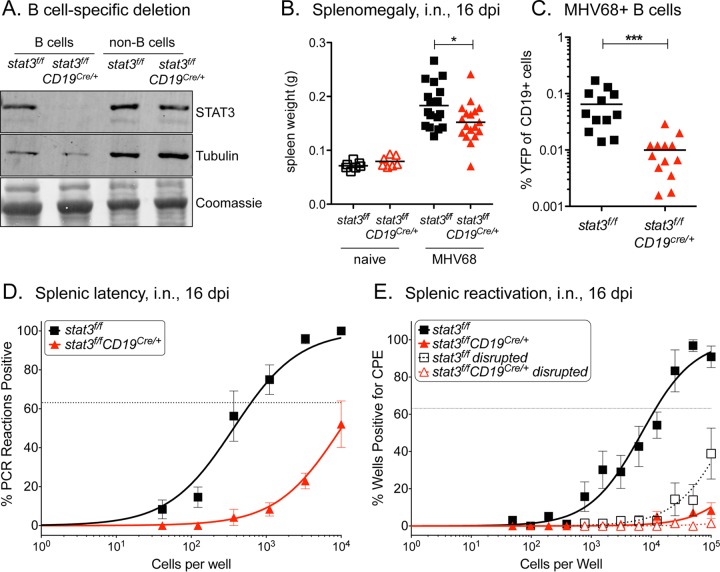
STAT3 is critical for the establishment of gammaherpesvirus latency in B cells. (A) Immunoblot of STAT3 from CD19^+^ B cell splenocytes of naive *stat3*^*f/f*^ and *stat3*^*f/f*^*CD19*^Cre/+^ mice sorted by flow cytometry to 96% and 86% purity, respectively. (B to E) *stat3*^*f/f*^ and *stat3*^*f/f*^*CD19*^Cre/*+*^ mice were infected with 1,000 PFU MHV68-YFP by intranasal (i.n.) inoculation and evaluated at 16 dpi. (B) Weights of spleens from uninfected and infected mice. Three independent experiments were performed with 3 to 7 mice per group. *, *P* < 0.05. (C) Evaluation of latency in B cells by flow cytometric evaluation of infected YFP^+^ CD19^+^ B cells. Two independent experiments were performed with 5 to 7 mice per group. ***, *P* ≤ 0.001. (D) Frequency of intact splenocytes harboring latent genomes. (E) Frequency of intact splenocytes that reactivated virus following explantation on fibroblasts. Dashed lines indicate disrupted splenocytes prior to quantification of preformed infectious virus. For panels B and C, each symbol represents an individual mouse. For the limiting dilution analyses whose results are shown in panels D and E, curve fit lines were determined by nonlinear regression analysis. Using Poisson distribution analysis, the intersection of the nonlinear regression curves with the dashed line at 63.2% was used to determine the frequency of cells that were positive for either the viral genome or the reactivating virus. Data are representative of the results of three independent experiments performed with 3 to 7 mice per group.

We addressed the impact of STAT3 on the ability of MHV68 to establish B cell latency by infecting mice with a tissue-specific deletion of STAT3 in B cells. Mice with a floxed STAT3 gene (*stat3*^*f/f*^) were crossed with mice expressing the Cre recombinase in B cells (CD19^Cre/+^) to drive the specific deletion of *stat3* in CD19^+^ B cells (36). Gene knockout efficiency was demonstrated by the absence of detectable levels of STAT3 expression in B cells isolated from splenocytes of *stat3*^*f/f*^*;CD19*^Cre/*+*^ mice (Fig. 1A).

To determine the role of STAT3 in viral latency, we infected *stat3*^*f/f*^*;CD19*^Cre/*+*^ mice and their littermate *stat3*^*f/f*^ controls with a recombinant MHV68 strain that encodes a yellow fluorescent protein (YFP) reporter gene (37). Intranasal infection with MHV68-YFP leads to an acute period of lytic replication in the nasopharynx and the lung, followed by rapid expansion of lymphocytes approximating infectious mononucleosis as the virus seeds lymphoid tissues. Within several weeks, replicating virus is cleared as the virus establishes a latent infection, with B cells serving as the predominant reservoir for latency. Splenic enlargement is a characteristic of MHV68 infection and reflects the simultaneous colonization of the spleen with MHV68 and the host response to this infection. At 16 days postinfection (dpi), the spleens of wild-type (WT) *stat3*^*f/f*^ mice increased 2-fold by weight, but, in comparison, there was a reduction in splenomegaly in the infected *stat3*^*f/f*^*;CD19*^Cre/*+*^ mice (Fig. 1B).

As a measure of viral colonization, the frequency of B cells that expressed the YFP viral reporter gene in the spleen at 16 dpi was determined by flow cytometry. There was a significant reduction in the percentage of YFP-positive (YFP^+^) B cells in the *stat3*^*f/f*^*;CD19*^Cre/*+*^ mice compared to the control *stat3*^*f/f*^ mice (0.065 ± 0.05% to 0.01 ± 0.008%, *P* = 0.007, respectively) (Fig. 1C). To determine the efficiency of viral latency establishment, the percentage of B cells that maintained the viral genome at 16 dpi was measured by limiting dilution of splenocytes and viral DNA PCR. Limiting dilution analysis of viral genome-positive cells enables the sensitive quantitation of intact cells that harbor the viral DNA. Approximately 1/623 splenocytes were positive for MHV68 in the control mice, while only 1/17,200 splenocytes were positive for MHV68 in mice lacking STAT3 in B cells (Fig. 1D and Table 1). Considering the reduction in splenomegaly with these results, the absence of STAT3 in B cells of *stat3*^*f/f*^*;CD19*^Cre/*+*^ mice reduced the total number of viral genome-positive splenocytes by 36-fold, from approximately 286,000 cells in the control mice to 7,910 cells in the *stat3*^*f/f*^*;CD19*^Cre/*+*^ mice (Table 1). This substantial reduction in latency was confirmed in an explant reactivation assay. The reactivation assay measures the frequency of intact splenocytes that can produce infectious virus when plated on monolayers of murine fibroblasts in tissue culture. Viral reactivation occurred at a rate of approximately 1/12,038 in the control *stat3*^*f/f*^ mice, but reactivation events were barely detectable in the *stat3*^*f/f*^*;CD19*^Cre/*+*^ mice (Fig. 1E). These data indicate that STAT3 is essential to promote efficient establishment of gammaherpesvirus latency in the B cells of the infected animal.

**TABLE 1:** Frequencies of cell populations harboring viral genomes

Mouse strain^a^	Route of infection^b^	Cell population^c^	dpi	Total no. of cells^d^	Frequency of viral-genome-positive cells^e^	Total no. of genome-positive cells^f^
*stat3^f/f^*	i.n.	Splenocyte	16	1.78 × 10^8^	1/623	286,000
	i.n.	Splenocyte	58	1.04 × 10^8^	1/13,300	7,840
						
*stat3*^*f/f*^*CD19*^Cre/+^	i.n.	Splenocyte	16	1.36 × 10^8^	1/17,200	7,910
	i.n.	Splenocyte	58	1.74 × 10^8^	1/79,400	2,190
						
*stat3*^*f/f*^	i.p.	Splenocyte	16	2.62 × 10^8^	1/117	2,060,000
	i.p.	CD19^+^ B cell	42	3.79 × 10^7^	1/3,950	9,590
						
*stat3*^*f/f*^*CD19*^Cre/+^	i.p.	Splenocyte	16	2.22 × 10^8^	1/785	284,000
	i.p.	CD19^+^ B cell	42	3.02 × 10^7^	1/31,530	958

a *stat3*^*f/f*^ mice are the WT *stat3* littermate controls corresponding to *stat3*^*f/f*^*CD19*^Cre/*+*^ mice that have a CD19^+^ B cell-specific deletion of *stat3*.

b i.n., intranasal; i.p., intraperitoneal.

c Data represent bulk unsorted splenocytes or the CD19^+^ B cell population isolated from spleens used for limiting dilution analysis.

d Data represent total numbers of the indicated cells isolated from the infected animals. Data represent means of results from cells recovered from two to three independent experiments performed with three to six individual mice per experiment.

e Data represent frequencies of the indicated cells that harbored the viral genome based on Poisson distribution analysis.

f Data were derived from the experimental frequency data and the approximate total number of cells per population.

### STAT3 is not required for gammaherpesvirus lytic replication.

**FIG 2:**
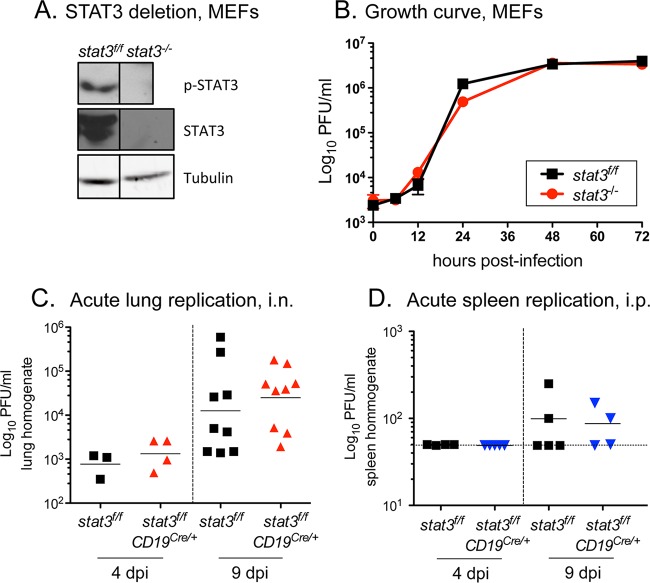
STAT3 is not essential for gammaherpesvirus replication. (A) Immunoblot of tyrosine-phosphorylated (p-STAT3) and total STAT3 protein in *stat3*^*f/f*^ and *stat3*^−/−^ murine embryonic fibroblasts (MEFs). Vertical lines indicate images joined together from the same blot to remove an independent clone. (B) Single-step growth curve in *stat3*^*f/f*^ and *stat3*^−/−^ MEFs at an MOI of 5.0 with WT MHV68-YFP. Symbols represent data from three independent wells ± standard deviations (SD). (C) Acute replication in the lungs of *stat3*^*f/f*^ and *stat3*^*f/f*^*CD19*^Cre/*+*^ mice infected with 1,000 PFU MHV68-YFP by intranasal inoculation at the indicated dpi. (D) Acute replication in the spleens of *stat3*^*f/f*^ and *stat3*^*f/f*^*CD19*^Cre/*+*^ mice infected with 1,000 PFU MHV68-YFP by intraperitoneal (i.p.) inoculation at the indicated dpi. Titers of lung and spleen homogenates were determined by plaque assay; the horizontal solid lines indicate the geometric mean titers. Each symbol represents an individual mouse. Dashed lines depict the limit of detection at 50 PFU/ml (log_10_ of 1.7).

To ensure that impairment of viral latency in the absence of STAT3 is not due to decreased acute viral replication, we first tested lytic replication in *stat3*^−/*−*^ murine embryonic fibroblasts (MEFs) devoid of STAT3 (Fig. 2A). There was no apparent change in the kinetics of viral replication in a single-step growth curve in the *stat3*^−/*−*^ MEFs compared to parental *stat3*^*f/f*^ MEFs (Fig. 2B) (38). Furthermore, following intranasal infection of the mice, acute viral replication and virus expansion in the lungs of *stat3*^*f/f*^*;CD19*^Cre/*+*^ mice were indistinguishable from the results seen in *stat3*^*f/f*^ mice (4 and 9 dpi) (Fig. 2C).

With an intraperitoneal route of infection, the spleen is another organ site of MHV68 replication. Although the levels of acute virus replication were lower in this organ at 4 and 9 dpi, the profile of replication following intraperitoneal administration of 1,000 PFU in the *stat3*^*f/f*^*;CD19*^Cre/*+*^ spleens was similar to the profile seen with *stat3*^*f/f*^ spleens (Fig. 2D). Increases of the dose to 10,000 and 100,000 PFU by the intraperitoneal route of infection led to higher levels of infectious virus in the spleen, but, again, the levels of acute replication seen in the *stat3*^*f/f*^*;CD19*^Cre/*+*^ and *stat3*^*f/f*^ mice were comparable (data not shown). The results indicate that STAT3 is not essential for MHV68 acute replication, and, more importantly, that the absence of STAT3 in B cells does not impact lytic expansion in the animal prior to colonization of the spleen.

### Deletion of *stat3* from B cells impairs establishment of gammaherpesvirus latency independently of the infection route.

**FIG 3:**
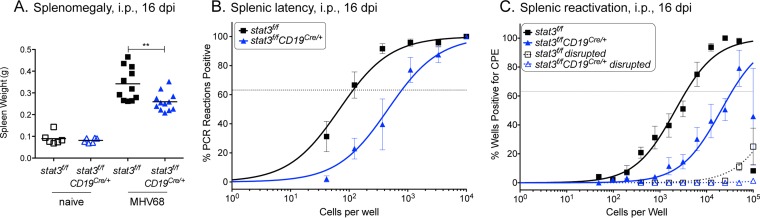
STAT3 is critical for the establishment of latency in B cells by the more direct intraperitoneal route of inoculation. *stat3*^*f/f*^ and *stat3*^*f/f*^*CD19*^Cre/*+*^ mice were infected with 1,000 PFU MHV68-YFP by intraperitoneal inoculation and evaluated 16 dpi. (A) Weights of spleens from uninfected and infected mice. Each symbol represents an individual mouse; 2 independent experiments were performed with three naive mice each, and three independent experiments were performed with 4 infected mice per group. **, *P* < 0.01. (B) Frequency of intact splenocytes harboring latent genomes. (C) Frequency of intact splenocytes undergoing reactivation from latency upon explantation. Dashed lines indicate splenocytes that were disrupted prior to plating to quantify preformed infectious virus. For the limiting dilution analyses whose results are shown in panels B and C, curve fit lines were determined by nonlinear regression analysis. Using Poisson distribution analysis, the intersection of the nonlinear regression curves with the dashed line at 63.2% was used to determine the frequency of cells that were positive for either the viral genome or the reactivating virus. Data represent compiled results from four independent experiments performed with 4 to 5 mice per group.

STAT3 clearly plays a critical role in the establishment of latency after intranasal infection that is not attributable to a defect in the expansion of the virus levels in the lungs (Fig. 1 and 2). Intranasal infection is commonly used as a route of infection for MHV68, since it most closely reflects a physiological relevant route of transmission. However, to understand the contribution of STAT3 to latency establishment and to eliminate the possibility that STAT3 influences B cell trafficking of the virus via the lymph nodes to the spleen, we evaluated the direct intraperitoneal route. Animals were infected with 1,000 PFU by intraperitoneal injection, and splenomegaly, viral latency establishment, and *ex vivo* viral reactivation were measured. The absence of STAT3 in B cells of infected mice again demonstrated a significant (*P* < 0.01) decrease in splenomegaly (Fig. 3A). Viral latency establishment at 16 dpi was measured by quantitation of viral genome-positive splenocytes. Limiting dilution of splenocytes and viral DNA PCR analysis showed a reduction from 1/117 cells in the control *stat3*^*f/f*^ mice to 1/785 in the *stat3*^*f/f*^*;CD19*^Cre/*+*^ mice (Fig. 3B and Table 1). The frequency of viral reactivation from latency following explantation was also reduced by ~10-fold (*P* = 0.017) (Fig. 3C). This reduction in latency after intraperitoneal inoculation demonstrates that STAT3 is a critical determinant in B cells for the virus to establish latency. Therefore, the viral latency defect observed after intranasal infection (Fig. 1) is not solely a failure in dissemination and trafficking from the site of acute replication in the lung to the spleen.

### B cell STAT3 is not required for germinal center processes during gammaherpesvirus infection.

Germinal centers in the spleen are sites of B cell activation, proliferation, immunoglobulin class-switch recombination, and plasma cell and memory cell differentiation. The gammaherpesviruses take advantage of B cell differentiation processes to gain access to immunoglobulin isotype class-switched memory B cells as a long-term latency reservoir (2, 39–44). A previous study of *stat3*^*f/f*^*;CD19*^Cre/*+*^ mice found a reduction in terminal plasma cell differentiation in response to the presence of a conjugated hapten (36). However, to the best of our knowledge, there have been no reports describing the STAT3-dependent B cell response to an active, dynamic virus infection.

**FIG 4:**
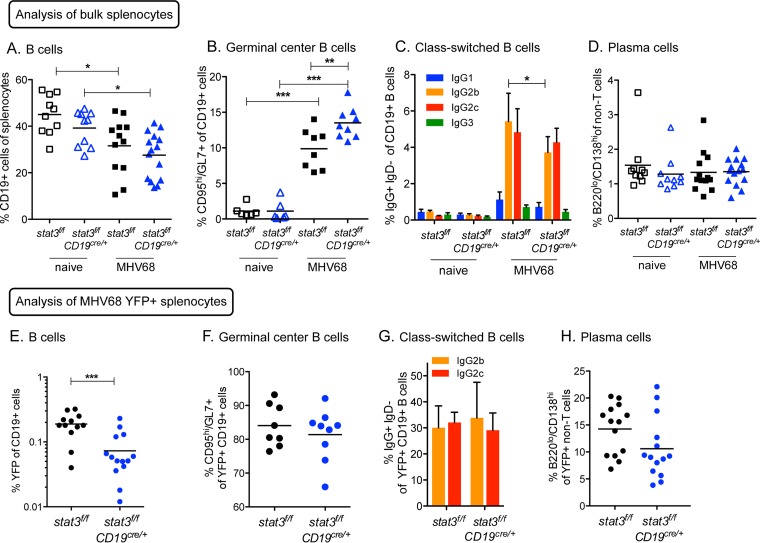
Loss of STAT3 in B cells does not impair virus colonization of germinal center and post-germinal-center B cells. *stat3*^*f/f*^ and *stat3*^*f/f*^*CD19*^Cre/*+*^ mice were infected with 1,000 PFU MHV68-YFP by intraperitoneal inoculation, and splenocytes were evaluated by flow cytometry at 16 dpi. (A) Frequency of CD19^+^ B cells of splenocytes gated on live lymphocytes from naive and infected mice. (B to D) Frequency of B cells that bear surface markers of germinal center B cells (GL7^+^ CD95^hi^), immunoglobulin class switching (IgG2b^+^ or IgG2c^+^ and IgD^−^), or plasma cells (B220^hi^ CD138^lo^). Each symbol represents an individual mouse. *, *P* < 0.05, **, *P* < 0.01, ***, *P* ≤ 0.001. (E) Frequency of B cells infected with MHV68 determined by percentage of CD19^+^ B cells that express the YFP marker. (F to H) Frequency of infected YFP^+^ B cells that bear surface markers of germinal center B cells (GL7^+^ CD95^hi^) (F), immunoglobulin class switching (IgG2b^+^ or IgG2c^+^ and IgD^−^) (G), or plasma cells (B220^hi^ CD138^lo^) (H). For panels A, D, E, and H, data represent compiled results from three individual experiments performed with 3 to 6 mice per group. For panels B and F, data represent compiled results from two individual experiments performed with 3 naive mice or 4 or 5 infected mice per group. For panels C and G, columns represent means ± SD of results from 4 to 6 individual mice.

To address the potential contribution of STAT3 to infected B cell maturation, proliferation, and differentiation, we examined the entire B cell population in the spleens of mice at 16 dpi, representing an active phase of the host adaptive immune response, 16 days after intraperitoneal infection. The percentages of B cells (CD19^+^) in the spleen with infection decreased similarly in the *stat3*^*f/f*^ and *stat3*^*f/f*^*;CD19*^Cre/*+*^ mice (Fig. 4A), consistent with the infiltration and proliferation of other cell types such as macrophages and T cells in response to gammaherpesvirus infection. Unexpectedly, the frequency of germinal center B cells (GL7^+^ CD95^hi^) in the infected *stat3*^*f/f*^*;CD19*^Cre/*+*^ mice was significantly increased in comparison to that seen with infected control *stat3*^*f/f*^ mice (Fig. 4B). To evaluate total B cell immunoglobulin class switching, we examined expression of IgG subclasses (Fig. 4C). In germinal center B cells, IgG2b and IgG2c were the predominant immunoglobulin isotypes in B cells of both *stat3*^*f/f*^ and *stat3*^*f/f*^*;CD19*^Cre/*+*^ mice, with a slight decrease in IgG2b levels upon the loss of STAT3 (*P* = 0.046) and no statistical difference in IgG2c levels. Terminal differentiation of B cells to plasma cells was found to occur at equivalent frequencies in the absence and presence of STAT3 (Fig. 4D). These analyses indicate that STAT3 is not essential in B cells for T cell-dependent germinal center formation or differentiation processes in response to intraperitoneal MHV68 infection.

In conjunction with the general profile of the B cell response to infection, we analyzed the stage-specific cell surface markers of infected B cells in the absence or presence of STAT3. As with the intranasal route of infection (Fig. 1C), flow cytometric analysis of MHV68 YFP-positive splenocytes from intraperitoneally infected animals revealed an approximately 3-fold (*P* < 0.001) reduction in the percentage of infected B cells that lacked STAT3 (*stat3*^*f/f*^*;CD19*^Cre/*+*^) compared to the levels seen with control *stat3*^*f/f*^ B cells (Fig. 4E). However, over 80% of the YFP-positive cells were identified as germinal center B cells (GL7^+^ CD95^hi^), regardless of the presence or absence of STAT3 (Fig. 4F). It is known that establishment of gammaherpesvirus latency in memory immunoglobulin isotype class-switched B cells supports long-term chronic infection (43, 44). For this reason, we examined the frequency of infected YFP-positive B cells with immunoglobulin surface receptors that had switched isotypes to IgG2b or IgG2c by flow cytometry. The distributions of YFP-positive B cells in either the IgG2b^+^ IgD^−^ or the IgG2c^+^ IgD^−^ subsets were similar in the control *stat3*^*f/f*^ and *stat3*^*f/f*^*;CD19*^Cre/*+*^ mice (Fig. 4G). Therefore, YFP-positive latent B cells that lack STAT3 are not impaired for isotype class switching.

Global loss of plasma cells by a B cell deletion of *prdm1* (BLIMP1) reduces establishment of MHV68 latency (42). Since STAT3 has been reported to upregulate *prdm1 in vitro* and to facilitate antibody production by class-switched B cells in the context of T-dependent antigen in mice (36, 45, 46), we evaluated the differentiation of plasma cells lacking STAT3 in the context of an ongoing MHV68 pathogen infection. While there was a noticeable trend for a decrease in the levels of YFP-positive plasma cells (B220^hi^ CD138^lo^) that lacked STAT3 (*stat3*^*f/f*^*;CD19*^Cre/*+*^), the mean values did not reach statistical significance in three independent experiments (Fig. 4H). Importantly, the lack of STAT3 did not phenocopy the absolute loss of plasma cells observed in the *prdm1*^*f/f*^;*CD19*^Cre/*+*^ mice. Taken together, STAT3 is required for MHV68 to establish latency in a significant portion of B cells, but this defect is not linked to a failure of infected B cells to participate in B cell differentiation processes initiating in the germinal center of secondary lymphoid tissues.

### Deletion of B cell STAT3 does not impair plasma cell function in response to MHV68 infection.

**FIG 5:**
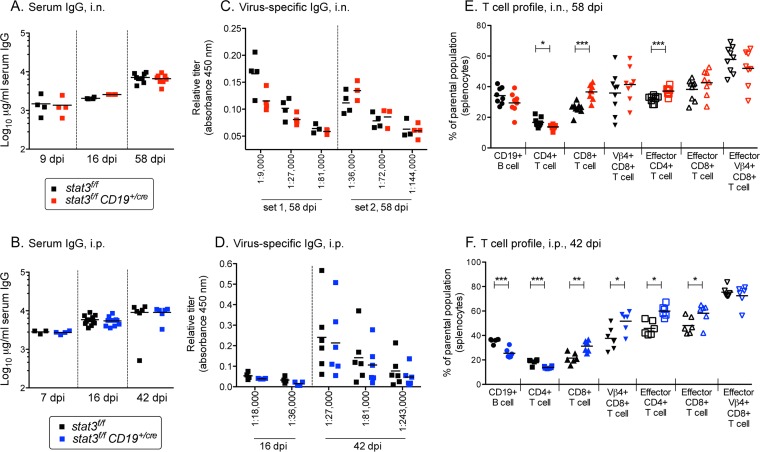
STAT3 is dispensable for adaptive immune responses to gammaherpesvirus infection. *stat3*^*f/f*^ and *stat3*^*f/f*^*CD19*^Cre/*+*^ mice were infected with 1,000 PFU MHV68-YFP by intranasal inoculation (A, C, and E) or intraperitoneal inoculation (B, D, and F) at the indicated dpi. (A and B) Total IgG production in sera of individual mice. The bars represent the geometric mean titers. (C and D) Relative titers of MHV68-specific IgG production in sera of individual mice at the indicated dilutions. (E and F) The T cell profile was evaluated by flow cytometric analysis for the frequency of splenocytes gated on live lymphocytes that bear surface markers of CD4^+^ T cells (CD4^+^ CD8^−^ CD19^−^), CD8^+^ T cells (CD8^+^ CD4^−^ CD19^−^), Vβ4^+^ CD8^+^ T cells (Vβ4^+^ CD8^+^ CD4^−^ CD19^−^) or effector CD4^+^ or CD8^+^ T cells (CD44^hi^ CD62L^lo^). Each symbol represents an individual mouse. *, *P* < 0.05, **, *P* < 0.01, ***, *P* < 0.001. For 42 and 58 dpi, data represent compiled results from two independent experiments performed with 3 to 5 mice per group.

To further determine a potential role of STAT3 in plasma cell differentiation and adaptive immune control in the context of infection with gammaherpesvirus, serum immunoglobulins were measured and T cell profiles were evaluated. Total IgG levels increased with the duration of infection similarly for *stat3^f/f^* and *stat3*^*f/f*^*;CD19*^Cre/*+*^ mice, regardless of whether the route of infection was intranasal or intraperitoneal (Fig. 5A and B). Most significantly, comparable levels of virus-specific IgG were generated in the *stat3*^*f/f*^ and *stat3*^*f/f*^*;CD19*^Cre/*+*^ mice at early and late times postinfection (Fig. 5C and D). The results indicate that STAT3 is not required for terminal plasma cell differentiation or immunoglobulin secretion in response to MHV68 infection.

CD4^+^ T cells and CD8^+^ T cells are critical components of the adaptive immune response and mediate long-term control of MHV68 during chronic infection (3, 47–52). Populations of Vβ4^+^ CD8^+^ T cells are dramatically expanded in C57BL/6 mice infected with WT MHV68, serving as an indicator of viral antigen expression and of the generation of T cell effector responses (53, 54). For this reason, we examined the T cell profile of mice by flow cytometry at 8 or 6 weeks after intranasal or intraperitoneal infection, respectively (Fig. 5E and F). There was a slight increase in the frequency of CD4^+^ T cells and CD8^+^ T cells in the *stat3*^*f/f*^*;CD19*^Cre/*+*^ mice compared to control *stat3*^*f/f*^ mice, regardless of the route of infection. However, the effector Vβ4^+^ CD8^+^ T cell populations expanded to comparable extents in the *stat3*^*f/f*^*;CD19*^Cre/*+*^ and *stat3*^*f/f*^ mice. Altogether, the data demonstrate that the production of antibodies or effector T cells in response to a chronic gammaherpesvirus infection does not require STAT3 in the B cell compartment.

### Deletion of STAT3 in B cells has a sustained negative impact on splenic latency.

**FIG 6:**
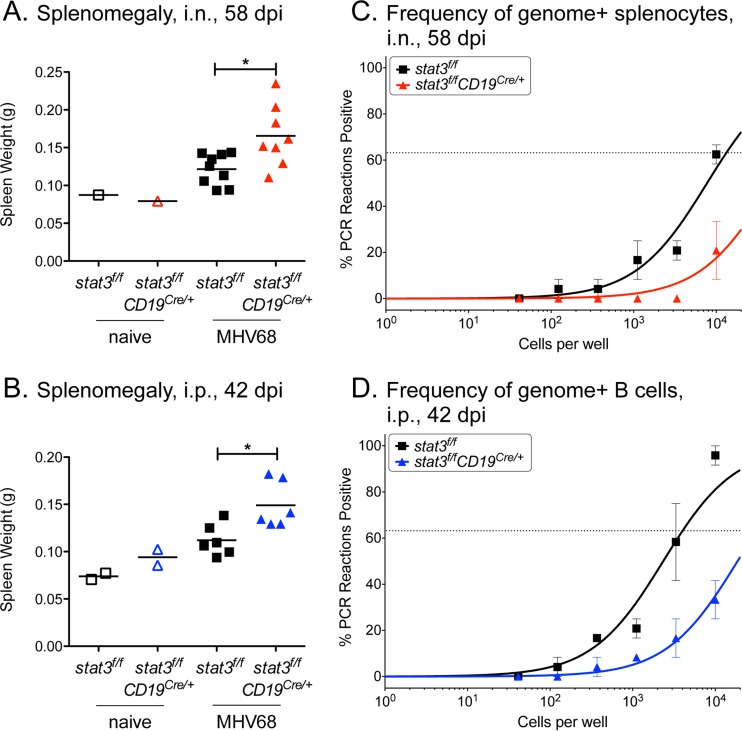
STAT3 loss in B cells has a sustained impact on splenic latency late during chronic infection regardless of the route of infection. *stat3*^*f/f*^ and *stat3*^*f/f*^*CD19*^Cre/*+*^ mice were infected with 1,000 PFU MHV68-YFP by intranasal inoculation and evaluated 58 dpi (A and C) or were infected by intraperitoneal inoculation and evaluated at 42 dpi (B and D). (A and B) Weights of spleens from uninfected and infected mice. Each symbol represents an individual mouse. *, *P* < 0.05. (C) Frequency of intact splenocytes harboring latent genomes by limiting dilution analyses. (D) Frequency of intact CD19^+^ B lymphocytes harboring latent genomes determined by limiting dilution analyses. B cells from *stat3*^*f/f*^ mice and *stat3*^*f/f*^*CD19*^Cre/*+*^ mice were enriched to 91% to 92% purity and 86% to 89% purity, respectively, by the use of magnetic beads. Curve fit lines were determined by nonlinear regression. Using Poisson distribution analysis, the intersection of the nonlinear regression curves with the dashed line at 63.2% was used to determine the frequency of cells that were positive for the viral genome. For the *stat3*^*f/f*^*CD19*^Cre/*+*^ mice, the last data point falls below 63.2%. Data represent compiled results from two independent experiments performed with 3 to 4 mice per group.

To determine if the role of STAT3 in latency establishment at 16 dpi extended into the maintenance phase of chronic infection, latency was examined 6 or 8 weeks after either intraperitoneal or intranasal infection, respectively. While splenomegaly of the infected mice was diminished at those later time points compared to 16 dpi, the infected spleens of the *stat3*^*f/f*^*;CD19*^Cre/*+*^ mice were significantly larger than those of *stat3*^*f/f*^ mice, regardless of the infection route (Fig. 6A and B). The increased splenomegaly was not due to an expansion in the B cell compartment (Table 1) but likely involved an increase in the levels of the T cell subsets that were observed by flow cytometry (Fig. 5E and F). The deletion of STAT3 in splenocytes had a sustained negative impact on viral latency following intranasal infection at the late time point of 58 dpi (Fig. 6C). The frequency of genome-positive splenocytes was diminished by approximately 6-fold in the *stat3*^*f/f*^*;CD19*^Cre/*+*^ mice (1/13,300) compared to the *stat3*^*f/f*^ mice (1/79,400) (Table 1). To verify that the defect in latency lay in the B cell reservoir, B cells were enriched from spleens 42 days after intraperitoneal infection. The frequency of viral latency in B cells that lacked STAT3 was ~1/31,530, an 8-fold reduction from the ~1/3,950 of B cells from the control mice (Fig. 6D and Table 1).

Taken together, these results indicate that MHV68 requires host STAT3 to establish B cell latency in infected animals and that this phenotype does not change with extended time. The loss of STAT3 does not appear to impair splenic B cell Ig class switching or differentiation to plasma cells that are able produce standard amounts of serum immunoglobulin (Fig. 4 and 5). We propose that STAT3 provides a critical function in supporting latency at the earliest stages of gammaherpesvirus engagement with the B cell that has a long-term impact on virus load in the host.

## DISCUSSION

The interface between virus and host is influenced by specific host gene expression that can steer the course of infection from nonpathogenic to pathogenic or from lytic to latent. In a murine model of pathogenesis, we identify STAT3 as a critical host determinant of gammaherpesvirus latency in B cells, a reservoir within the microenvironment of secondary lymphoid tissues awash in cytokines known to engage JAK/STAT signaling and activate STAT3 (55). The MHV68 murine model provides a dynamic system to follow long-term latency establishment in the B cell compartment of the host, similarly to human gammaherpesviruses. To determine the contribution of STAT3 to gammaherpesvirus latency in B cells during the course of a dynamic infection, we evaluated animals with a *stat3* deletion in B cells. Using this genetic system with a loss of STAT3 confined to CD19^+^ B cells, we found a substantial reduction in the levels of MHV68 latent DNA during chronic infection.

Plasma cell development in *stat3*^*f/f*^*CD19*^Cre/+^ mice appears to be intact. MHV68 is a large DNA virus that provides a myriad of antigenic epitopes, and the levels of virus-specific antibodies and the expansion of the T-cell response were not impaired in the *stat3*^*f/f*^*CD19*^Cre/+^ mice following infection. MHV68 reactivation from latency at the early stage of chronic infection has been shown to involve the BLIMP1 transcription factor and its ability to drive terminal plasma cell differentiation (42, 94). STAT3 contributes to BLIMP1 expression and plasma cell differentiation in coordination with IRF4 following IL-21 stimulation in murine B cells (45, 46). Strikingly, we did not observe a defect in germinal center or plasma cell formation in *stat3*^*f/f*^*CD19*^Cre/+^ mice. We also did not find evidence for persistent viral replication in the lungs of *stat3*^*f/f*^*CD19*^Cre/*+*^ mice at 8 weeks postinfection (data not shown), an effect previously noted in *CD40*^−/*−*^ mice and *p50*^−/*−*^ mice that fail to generate cellular and humoral immune responses (56, 57). Taking the data together, B cell STAT3 does not appear to be essential for terminal plasma cell IgG production or virus reactivation in the context of a complex and chronic gammaherpesvirus infection. Similarly, memory B cells from hyper-IgE patients with a spectrum of *stat3* loss-of-function mutations were not impaired for plasmablast generation upon cytokine stimulation in culture (58). Thus, other known transcription factors that regulate BLIMP1 to drive B cell differentiation likely compensate for STAT3 loss (59).

Mutations in the DNA-binding or transactivation domain of STAT3 in hyper-IgE syndrome patients are known to negatively impact immune cell function and exacerbate infectious disease and EBV lymphomas (60–64). Since these STAT3 mutations are expressed in all the tissues of the patients, the global impact of the deficiency is profound and complex (65). Even with this proviso, B cells isolated from these patients are unable to support EBV-driven outgrowth (66). In tissue cell culture studies, STAT3 has been reported to contribute to the expression of EBV latency genes (18, 67–69) and to promote EBV- and KSHV-mediated cell proliferation (14, 70). STAT3 activation is associated with latent KSHV infection of endothelial cells (71) and chromatin remodeling that facilitates the latent gene expression program of herpes simplex virus 1 in neurons (72). Thus, STAT3 may serve a dual role in the promotion of a latent gene expression program in newly infected B cells while also promoting proliferation and other effector functions of the cell. These studies illustrate the complex interactions of herpesviruses with the JAK/STAT signaling pathway.

B cells are exposed to cytokines that are known to engage JAK/STAT signaling and activate STAT3 in the lymphoid tissue of MHV68-infected animals (55). Most cytokine pathway knockout studies do not use tissue-specific conditional deletions; as a result, all the cells of the animal lack the specified gene, causing systemic effects. The influence of genetic knockout of several of these cytokines or cytokine receptors has been evaluated in the context of MHV68 pathogenesis, and, notably, the phenotypes of those knockouts are distinct from those seen with the *stat3*^*f/f*^*;CD19*^Cre/*+*^ mice reported here. Mice lacking interferon (IFN) gamma or the IFN gamma receptor exhibited increased levels of viral latency and reactivation in splenocytes and even enhanced reactivation from the macrophage compartment following MHV68 infection (50, 73). IL-6, a classical cytokine activator of STAT3, was found to be dispensable for viral latency and cytotoxic T cell responses to MHV68 infection (74–76).

Two cytokines that activate STAT3 and appear to influence murine gammaherpesvirus latency are IL-10 and IL-21. IL-10 stimulates STAT3 and promotes B cell survival and yet is suppressive for inflammation and impairs T cell control of MHV68 (77–80). IL-10 knockout mice have reduced viral reactivation from splenocytes but an increase in splenic weight and leukocytosis at 15 dpi (81), unlike the infected *stat3*^*f/f*^*;CD19*^Cre/*+*^ mice (Fig. 1). Murine gammaherpesvirus colonization of germinal center B cells is a process driven in large part by IL-21 production by T follicular helper cells (33). IL-21 receptor knockout animals have a defect in latency establishment following intranasal MHV68 infection, but this is linked to a loss of germinal center formation, IgG class switching, and plasma cell differentiation (33). Mixed bone marrow chimera experiments demonstrated that the failure to support latency and participate in germinal center reactions is intrinsic to B cells that cannot sense IL-21 (33). Our infections of the *stat3*^*f/f*^*CD19*^Cre/*+*^ mice by the intraperitoneal route did not reveal a generalized defect in germinal center participation or IgG class switching for B cells lacking STAT3. Viral latency was clearly impaired in the STAT3-null B cells, but, among the cells that were infected, there was no blockade in germinal-center-dependent B cell differentiation processes (Fig. 4F to H). The effects of a single cytokine or a cytokine receptor knockout in the microenvironment of the spleen may be compensated for by other cytokines to some degree. Future pathogenesis studies of receptor knockouts crossed to the *stat3*^*f/f*^*;CD19*^Cre/*+*^ mice may determine if STAT3 is the key downstream host factor required for B cell-intrinsic responses to specific cytokines to support gammaherpesvirus latency.

We observed a relative decrease in the percentage of splenic B cells that corresponded to an increase in levels of CD4^+^ T cells and CD8^+^ T cells at 6 to 8 weeks after infection of the B cell-specific *stat3*-deleted mice, regardless of the route of infection (Fig. 5). The reduction in the viral load at those late times may be partially attributable to an increase in effector T cell function. T cell responses would be expected to increase in the context of antigen exposure. However, the deletion of *stat3* reduced viral latency and there was no increase in reactivation from the spleen or peritoneal cavity and no evidence of recrudescence in the lungs (data not shown). Perhaps STAT3 functions to repress sporadic reactivation events during chronic infection that result in viral antigen presentation. Another consideration is the role of STAT3-regulated immunosuppressive cytokine IL-10 in suppressing T cell responses and driving regulatory T cell differentiation (82–85). IL-10 dampens T cell responses to MHV68 infection (80, 86). Since IL-10 production is driven by the viral M2 gene product in MHV68-infected B cells, a reduction in latency at 16 dpi might reduce IL-10 levels (86, 87). In addition, disregulation of the IFN-alpha/pSTAT3/IL-10 axis in systemic lupus erythematosus patients has revealed that the levels of type 1 IFN produced by plasmacytoid dendritic cells dictate the formation of regulatory B cells in a STAT3-dependent manner (88). Future studies are needed to determine whether STAT3 deletion in B cells influences IL-10 production and T cell responses during gammaherpesvirus infection.

Our study was the first to demonstrate the impact of a targeted deletion of the STAT3 transcription factor specifically in B cells, the primary latency reservoir for murine gammaherpesvirus. Given the B cell-intrinsic phenotype in the mouse model, we are now poised to dissect the specific contributions of STAT3 that enable a gammaherpesvirus to establish a latent gene expression program *in vivo*. STAT3 may directly regulate MHV68 gene expression or indirectly promote a cellular environment conducive to B cell latency. MHV68 targets transitional B cells and marginal-zone B cells (89–91), and STAT3 might play a critical role in the infection of these subsets prior to germinal center entry. The defects apparent in the early period of latency establishment were not ameliorated with time, indicating that STAT3 is a critical determinant of both the establishment and the maintenance of latency in the infected host. While many STAT3-responsive genes have been identified in transformed cells, the contribution of STAT3 to B cell biology and gammaherpesvirus latency in the host is poorly understood. Future studies to identify target host and viral genes in the infected B cells that require STAT3 to support gammaherpesvirus latency may provide understanding that will identify novel therapeutics that can be tested in the murine model.

## MATERIALS AND METHODS

### Mice and cells.

*stat3*^*f/f*^ mice (38) and CD19-*Cre* mice [B6.129P2(C)-Cd19 ^tm1(cre)Cgn^/J; The Jackson Laboratory, Bar Harbor, ME] were bred at the Stony Brook University Division of Laboratory Animal Research facility. All protocols were approved by the Institutional Animal Care and Use Committee of Stony Brook University. The *stat3*^*f/f*^ and *stat3*^*f/f*^*CD19*^Cre/+^ mice used in all pathogenesis experiments were littermates derived from crossing *stat3*^*f/f*^ mice with *stat3*^*f/f*^ mice heterozygous for *CD19-Cre*. Immortalized *stat3*^*f/f*^ and *stat3*^−/−^ MEFs were as described previously (92).

### Infections and organ harvests.

MHV68 expressing the H2B-YFP protein (MHV68-YFP) was used for the infections (37). WT mice (8 to 12 weeks old) were either infected by intranasal inoculation with 1,000 PFU MHV68-YFP in 20 µl or by intraperitoneal injection with 1,000 PFU in 0.5 ml under conditions of isoflurane anesthesia. Mice were sacrificed by the use of terminal isoflurane anesthesia. For determination of acute titers, mouse lungs or spleens were harvested and stored at −80°C prior to disruption in a Mini-BeadBeater (BioSpec, Bartlesville, OK). The titers of the homogenates were determined by plaque assay. For latency, reactivation, and flow cytometry experiments, mouse spleens were homogenized, treated to remove red blood cells, and passed through a 100-µm-pore-size nylon filter.

### Immunoblot analysis.

Total protein lysate was harvested in lysis buffer (150 mM sodium chloride, 1.0% IGEPAL CA-630, 0.5% sodium deoxycholate, 0.1% sodium dodecyl sulfate, 50 mM Tris [pH 8.0]) supplemented with a protease inhibitor cocktail (Sigma, St. Louis, MO) and phenylmethylsulfonyl fluoride (PMSF). Proteins were separated on 10% SDS-PAGE gels and transferred to a polyvinylidene fluoride membrane. Antibodies against STAT3 (K-15; Santa Cruz Biotechnology, Dallas, TX), tyrosine 705-phosphorylated STAT3 (Cell Signaling Technology, Danvers, MA), and antitubulin (clone B-5-1-2; Sigma) were detected by the use of secondary anti-mouse (Rockland, Limerick, PA) or secondary anti-rabbit (Invitrogen, Grand Island, NY) antibodies by immunoblot analysis with an Odyssey Imager (Li-COR Biosciences, Lincoln, NE).

### Virus replication.

*stat3*^*f/f*^ and *stat3*^−/−^ MEFs were seeded in triplicate in 6-well plates, and cells were infected the following day at a multiplicity of infection (MOI) of 5 PFU per cell for single-step replication kinetics. The cells and conditioned medium were freeze-thawed four times, and NIH3T12 fibroblast cells were infected with serial dilutions of the samples for 1 h and then overlaid with a 1.5% methylcellulose solution in Dulbecco’s modified Eagle’s medium (DMEM) supplemented with 5% fetal bovine serum (FBS). One week later, the monolayers were washed with phosphate-buffered saline (PBS) and fixed in 100% methanol and plaques were visualized by staining using 0.01% crystal violet solution containing 20% methanol.

### Limiting dilution analysis of latency and reactivation.

To determine the frequency of cells harboring the viral genome, single-cell suspensions were prepared for single-copy nested PCR. In brief, six 3-fold serial dilutions of intact cells were plated on NIH 3T12 cells and lysed overnight at 56°C with proteinase K. The samples were subjected to an 80-cycle nested PCR with primers specific for MHV68 ORF50 (93). A total of 12 replicates were analyzed at each serial dilution, and plasmid DNA was included at 0.1, 1.0, and 10 copies as a control. To determine the frequency of cells harboring latent virus capable of reactivation upon explantation, single-cell suspensions were plated in 12 serial 2-fold dilutions on a monolayer of MEF cells prepared from C57BL/6 mice. A total of 24 replicates were plated per serial dilution. Cytopathic effect (CPE) was scored 2 and 3 weeks after plating. To differentiate between preformed infectious virus and virus spontaneously reactivating upon cell explantation, parallel samples were mechanically disrupted using a Mini-BeadBeater prior to plating on the monolayer of MEFs to release preformed virus and the results were scored as CPE (93).

### Flow cytometry.

For the analysis of B cells, 2 × 10^6^ splenocytes were resuspended in 200 µl of fluorescence-activated cell sorter (FACS) buffer (PBS with 2% fetal bovine serum) and blocked with TruStain fcX (clone 93; BioLegend, San Diego, CA). The cells were washed and stained to identify B cell subsets with fluorophore-conjugated antibodies against CD19 (clone 6D5), B220 (clone RA3-682) CD138 (clone 281-2), CD95 (clone 15A7), and IgD (clone 11-26c.2a) or biotinylated antibodies against GL7 (clone GL-7), IgG1 (clone RMG1-1), IgG2b (clone R12-3), IgG2c (clone RMG2a-62), or IgG3 (clone R40-82) that were detected by the use of secondary streptavidin-conjugated allophycocyanin. T cell subsets were identified with antibodies against CD4 (clone GK1.5), CD8 (clone 53-6.7), Vβ4 (clone KT4), CD44 (clone IM7), and CD62L (clone MEL-14). For sorting, splenocytes were first enriched for B cells by depletion of non-B cells with magnetic microbeads (Pan B cell isolation kit; Miltenyi Biotec, Auburn, CA) and then sorted using CD19 conjugated to phycoerythrin and a FACSAria III sorter (BD Biosciences). All antibodies were purchased from BioLegend or BD Biosciences (San Jose, CA). The data were collected using a Dxp-8 FACScan flow cytometer (Cytek Development, Fremont, CA) and analyzed using FlowJoX v10.0.7 (Treestar Inc., Ashland, OR).

### Enzyme-linked immunosorbent assay (ELISA).

To measure total IgG levels in the serum, plates were coated with 2 µg/ml of donkey anti-mouse IgG (Affinipure; Jackson ImmunoResearch Laboratories, West Grove, PA)–PBS, washed in PBS with 0.05% Tween 20, and blocked in 1% bovine serum albumin (BSA)–PBS prior to incubation with serial dilutions of serum or the mouse IgG standard (ChromPure; Jackson ImmunoResearch) in assay diluent (OptEIA assay diluent; BD Biosciences) overnight at 4°C. IgG was detected by the use of horseradish peroxidase-conjugated donkey anti-mouse IgG (Jackson ImmunoResearch), R & D Systems (Minneapolis, MN) substrate, and stop solution, and absorbance at 450 nm was read on a FilterMax5 microplate reader (Molecular Devices, Sunnyvale, CA). To measure levels of IgG specific for viral antigens, plates were first coated with 0.5% paraformaldehyde-fixed viral antigen–PBS overnight at 4°C.

### Statistical analyses.

Data were analyzed using GraphPad Prism software (Prism 5; GraphPad Software, Inc., La Jolla, CA). Statistical significance was determined using either analysis of variance (ANOVA) followed by a Tukey’s posttest or a nonpaired two-tailed *t* test. Using Poisson distribution analysis, the frequencies of latency establishment and reactivation from latency were determined by the intersection of nonlinear regression curves with the line at 63.2%. Significance was evaluated by paired one-tailed *t* tests of the log-transformed frequency values of samples from matched experiments.
